# An assessment of the geographical risks of wild and vaccine-derived poliomyelitis outbreaks in Africa and Asia

**DOI:** 10.1186/s12879-017-2443-4

**Published:** 2017-05-26

**Authors:** Kathleen M. O’Reilly, Christine Lamoureux, Natalie A. Molodecky, Hil Lyons, Nicholas C. Grassly, Graham Tallis

**Affiliations:** 1 MRC Centre for Outbreak Analysis and Modelling, Department of Infectious Disease Epidemiology, St Mary’s Campus, Imperial College London, London, UK; 2 World Health Organization, Geneva, Switzerland; 3 Institute for Disease Modelling, Seattle, USA

**Keywords:** Poliomyelitis, Risk assessment, Regression, Outbreak, Vaccine-derived, Routine immunisation, Vaccination, Migration

## Abstract

**Background:**

The international spread of wild poliomyelitis outbreaks continues to threaten eradication of poliomyelitis and in 2014 a public health emergency of international concern was declared. Here we describe a risk scoring system that has been used to assess country-level risks of wild poliomyelitis outbreaks, to inform prioritisation of mass vaccination planning, and describe the change in risk from 2014 to 2016. The methods were also used to assess the risk of emergence of vaccine-derived poliomyelitis outbreaks.

**Methods:**

Potential explanatory variables were tested against the reported outbreaks of wild poliomyelitis since 2003 using multivariable regression analysis. The regression analysis was translated to a risk score and used to classify countries as Low, Medium, Medium High and High risk, based on the predictive ability of the score.

**Results:**

Indicators of population immunity, population displacement and diarrhoeal disease were associated with an increased risk of both wild and vaccine-derived outbreaks. High migration from countries with wild cases was associated with wild outbreaks. High birth numbers were associated with an increased risk of vaccine-derived outbreaks.

**Conclusions:**

Use of the scoring system is a transparent and rapid approach to assess country risk of wild and vaccine-derived poliomyelitis outbreaks. Since 2008 there has been a steep reduction in the number of wild poliomyelitis outbreaks and the reduction in countries classified as High and Medium High risk has reflected this. The risk of vaccine-derived poliomyelitis outbreaks has varied geographically. These findings highlight that many countries remain susceptible to poliomyelitis outbreaks and maintenance or improvement in routine immunisation is vital.

**Electronic supplementary material:**

The online version of this article (doi:10.1186/s12879-017-2443-4) contains supplementary material, which is available to authorized users.

## Background

Since the commitment to eradicate poliomyelitis in 1988 there has been a substantial reduction in the number of cases of poliomyelitis, and poliovirus has been eliminated from a majority of countries. India had successfully eliminated wild poliomyelitis in 2014 [1], resulting in considerable reduction in risk within the local population and neighbouring countries. Following an absence of cases for over 2 years, wild poliomyelitis was detected in Borno State, Nigeria, in August 2016, where genetic sequencing of the virus suggests undetected transmission since at least 2013. Endemic transmission continues in Pakistan and Afghanistan, but here the incidence of wild poliomyelitis cases has reached an all-time low [1].

Successful elimination of wild poliomyelitis from India and substantial reduction in incidence in Nigeria was the result of considerable efforts to immunise large numbers of children each year, primarily through use of supplementary immunisation activities (SIAs). Transmission of poliomyelitis in Nigeria and India prior to this time resulted in several outbreaks of poliomyelitis in countries previously free from disease. The large number of outbreaks, resulting in the re-establishment of transmission in several countries, was a cause of great concern [2]. Since this time there has been a refocus in resources and planning of preventive SIAs, and development of country-specific response plans [3].

A previous analysis of wild poliomyelitis outbreaks had identified that population immunity, recent outbreaks, extent of travel from infected countries and factors such as the percentage of the population aged less than 15 years were associated with poliomyelitis outbreaks [4]. This analysis set out a framework where preventive SIAs could be planned based on inference from a statistical model that captured the different contributions of population immunity, migration from affected countries and additional factors, rather than relying solely on expert assessment of risk. To this end, a multi-agency team representing partners of the Global Polio Eradication Initiative was established in 2014 to support SIA planning by providing an assessment of wild poliomyelitis risk [5]. In addition to this international assessment of risk, WHO regional offices make sub-national assessments of poliomyelitis risk [6].

An inevitable consequence associated with the use of the live attenuated oral polio vaccine (OPV) is the emergence of vaccine-derived polioviruses (VDPVs), which in some situations has led to outbreaks of poliomyelitis [7]. The first outbreak of VDPV was reported in Hispaniola in 2000. Several studies have explored the mechanisms leading to VDPV emergence; the attenuated poliovirus strains used to produce the OPV naturally mutate and these revertants with increased neurovirulence are naturally selected during replication in the gut of OPV recipients (sometimes resulting in cases of vaccine-associated paralytic poliomyelitis [8]). Should this occur in areas of low population immunity the revertants may spread within populations, resulting in cases of paralytic poliomyelitis where the isolated virus may have >1% nucleotide sequence divergence (0.6% for serotype 2) from the original Sabin strain, indicating ongoing circulation [7]. Within Nigeria, districts with low routine immunisation coverage and a high number of births were associated with a high probability of VDPV emergence and circulation [9]. To eliminate the risks posed by circulating VDPVs (cVDPVs) of serotype 2, OPV of this serotype has been withdrawn from immunisation activities through a globally synchronised replacement of all trivalent OPV with bivalent OPV and inclusion of at least one dose of the inactivated poliovirus vaccine in the routine immunisation schedule [10]. In the long-term, withdrawal of serotype 2 from the OPV will probably reduce the overall risks of cVDPV2s, but outbreaks from serotypes 1 and 3 may still occur. To minimise the risks associated with VDPVs while OPV is still in use, it is important to understand the geographical variation in risk and identify suitable measures to limit emergence.

A challenge associated with modelling of infectious diseases is integration of research findings into public health policy [11]. A mutually-beneficial, often long-standing relationship between policy makers and epidemiological modellers is necessary for modelling inference to be successfully translated into policy, where the timeliness and interpretability of modelling results are critical to be useful. Here we describe a risk scoring method that has been used to inform an international poliomyelitis risk assessment since 2014 and can be rapidly applied to additional countries or additional time-frames. The methodology is also applied to cVDPV risk and the spatial and temporal variation in risk associated with wild and cVDPV outbreaks are compared.

## Methods

### Description of the data

Outbreaks of poliomyelitis were identified through global surveillance of acute flaccid paralysis (AFP) and testing of stool specimens at WHO-accredited laboratories of the Global Polio Laboratory Network [1]. The initial clinical symptoms of poliomyelitis are characterised by the rapid onset of weakness, typically but not exclusively in the lower limbs, with involuntary muscle paralysis but no loss of sensation [12]. Isolation of the virus from stool confirms poliomyelitis as opposed to other (infectious and non-infectious) causes. Stool samples from all suspect cases and/or contacts are screened for the presence of poliovirus, where real-time polymerase chain reaction assays and sequence analysis are used to determine the serotype and whether the isolated virus is of wild or vaccine-derived origin [13, 14]. If multiple poliomyelitis cases of the same genotype were identified within a country, cluster analysis was used to determine whether cases were a result of independent emergences or transmission. For VDPVs an isolate was designated as cVDPV if more than 1% (0.6% for serotype 2) nucleotide divergence was apparent and there was evidence of transmission (more than one related poliomyelitis case), consistent with definitions developed by the Centers for Disease Control [13]. The definition of a cVDPV has changed since July 2015 to include more sensitive conditions for detecting circulation [15], but for consistency here we use the old definition. The number, timing and country of origin of cVDPV outbreaks were compiled from Centers for Disease Control reports from 2003 to 2016 (Additional file 1), where country outbreaks from cross-border transmission (and identified through sequence analysis) were excluded. In some instances, cVDPV outbreaks may have been a result of one or more emergent events and the six-month time period was classified as an emergent event occurring rather than the exact number of events. The analysis was restricted to countries within the AFRO, EMRO and SEARO regions and selected countries in the EURO region (Kazakhstan, Kyrgyzstan, Tajikistan, Turkey, Turkmenistan, Uzbekistan, Ukraine).

Surveillance for poliomyelitis captures all reported cases of AFP where case investigation includes parental recall of the number of OPV doses from routine immunisation and SIAs. The dose histories of non-polio AFP cases from children aged 6–59 months recorded from 1 January 2003 to 28 July 2016 were used to estimate the percentage of children under 5 years who were under-immunised (received less than 3 OPV doses) and the percentage of children who report zero OPV doses. Statistical models were employed to provide country-level, temporally-smoothed estimates in bins of six months for the percentages of under-immunised and zero-dose children. The models used first order-random walks to model the temporal changes in the percentages along with country level random intercepts to model overall differences between countries [16]. The smoothed data were especially relevant for small countries with limited numbers of non-polio AFP cases. These estimates of immunisation performance were used as indicators of population immunity instead of estimating the percentage of the population protected from paralysis [17], as the vaccine efficacy of the OPV is known to vary between settings and estimates are only available from a limited number of countries.

Indicators of OPV immunisation via routine services were included in the analysis by using national estimates of the third dose of the diphtheria-tetanus-pertussis DTP3 vaccine, which is representative of three doses of the OPV [18]. Other potential risk factors were hypothesised based on expert opinion and previously published research [6] (Table 1). Exposure to poliovirus associated with population movement from countries with circulation of wild poliovirus is well-known to be a risk factor for international spread [4, 19], and several measures of population movement were tested in the regression analysis. Exposure to poliovirus experienced by country j per six-month time-period (*λ*_*jt*_) was estimated from data of economic migration [20] and refugees [21] and poliomyelitis cases in country *i* in the previous six months (*x*_*i*, t − 1_) using ${\lambda}_{jt}=\mathit{\log}\sum_{i=1}^n{m}_{i j}{x}_{i, t-1}$, where t is the binned time period, *m* is the measurement of migration and *n* is the total number of countries included in the analysis, as in [4].

**Table 1:** Variables tested in the regressions models that were tested for an association with wild and VDPV outbreaks

Variable	Description	Data source	Continuous/discrete	Wild outbreaks	cVDPV outbreaks
Population immunity					
Under-immunised	Smoothed % of children under 5 yo with >2 OPV doses	AFP	Both	Y	Y
Zero-dose	Smoothed % children under 5 yo with 0 OPV doses	AFP	Both	Y	Y
Routine immunisation	% under 1 yo that received 3+ doses of the DTP vaccine	WHO/Unicef	Both	Y	Y
Surveillance quality					
Stool surveillance	% AFP cases under 15 yo with adequate^a^ stool samples processed	AFP	Discrete	Y	Y
npAFP rate	Number of non-polio AFP cases per 100,000 population under 15 yo	AFP	Discrete	Y	Y
Historical propensity					
Wild-type outbreaks	Number of wild-type outbreaks reported in last 4 years	WHO	Both	Y	Not tested
Wild-type multi-case outbreaks	Number of wild-type outbreaks with >1 case reported in last 4 years	WHO	Both	Y	Not tested
cVDPV outbreaks	Number of VDPV outbreaks reported in last 4 years			Not tested	Y
cVDPVs multi-case outbreaks	Number of VDPV outbreaks with >1 case reported in last 4 years			Not tested	Y
Migration					
Proximity	Wild-type outbreak present in a bordering country in last 6 months	Cluster description	Discrete	Y	Not tested
Proximity scaled	The number of poliomyelitis cases in bordering countries in previous six months	Cluster description	Continuous	Y	Not tested
Migrants	Number of migrants multiplied by incidence within country in previous 6 months	www.migrationdrc.org	Both	Y	Not tested
Refugees	Number of refugees multiplied by incidence within country in previous 6 months	UNHCR	Both	Y	Not tested
Humanitarian concern					
Displaced	% total population registered as refugees and internally displaced persons	UNHCR	Discrete	Y	Y
Other events (Ebola)	Adverse humanitarian events that have occurred in the previous 4 years	Expert opinion	Discrete	Y	Y

^a^Adequate stools refer to two samples from AFP cases being collected ≥24 h apart, both within 14 days of paralysis onset

International population movements rapidly change according to economic drivers, political instability, natural disasters and on occasion infectious diseases (such as Ebola), where especially in low-income settings the scale of movement is inconsistently documented or is unrecorded. To account for population movements in addition to that recorded (for example those reported by epidemiological field investigators), expert opinion was also incorporated into the risk assessment. The movement patterns were assessed using a “likelihood-versus-consequence” matrix [22], which is commonly used in qualitative risk assessments (see Additional file 1). Information on population movements from each country were ranked according to the perceived likelihood of the movements increasing the risk of virus transmission across borders and the impact of such increased transmission within the country of destination. Movements assessed as high risk were allocated a score of 1 in the risk assessment.

### Statistical analysis

A mixed-effects logistic regression model was used to identify factors associated with one or more wild poliomyelitis outbreaks reported by a country or region for every six months of the study period (1 January 2003 to 30 June 2016). The regression model consists of an intercept (*β*_0_), fixed (*β*) and random variables (*b*_*i*_), ie. *logit*(*E*(*Y*_*i* ,t+ 1_| *β*_0_, *β*, *b*_*i*_)) = *β*_0_ + *X*_*i* , *t*_ *β* + *b*_*i*_. Explanatory variables significant (*p* < 0.2) in the univariable analysis were tested in the multivariable model. In the multivariable model variables were selected if a chi-squared test illustrated a significant (*p* < 0.05) association between the variable and the outcome, and if the Akaike’s information criteria reduced in value when compared to the reduced model. Interactions between model variables were tested, and in some cases, risk factors were still included in the final model even if the *p*-values were greater than 0.05. Regression analysis was also carried out to test for associations with the emergence of cVDPVs of all serotypes, again using a mixed-effects logistic regression model. As cVDPV emergence is a function of exposure to OPV which is largely driven by births and associated routine immunisation activities [9], the annual number of births for each country was forced into the model. For the regression analysis birth numbers were included on a natural log scale (which is analogous to an offset term), and grouped into three categories; <500,000, ≥500,000 to <1,000,000 and ≥1,000,000.

The coefficients of the final multivariable model were used to develop a risk score. The score was calculated by scaling the coefficients by the smallest coefficient of the final risk model and rounding to the nearest integer [23]. Variables on a continuous scale were grouped into categories and the regression coefficient for the categorical variables were used to calculate the risk score. Country-level random effects were excluded. For compilation with the other agency risk models, the risk score was converted to four categories: Low, Medium, Medium High and High. The translation of the risk score to the categories was made by balancing the sensitivity, specificity and probability of experiencing an outbreak.

The regression analysis of the probability of reporting a poliomyelitis outbreak included only variables from the previous time periods, enabling six-month ahead forecasts of the probability of reporting an outbreak in a country. The predictions of each model were compared to the outbreaks reported in the subsequent time-period. Previous analysis had used “area under the curve” diagnostics [4], but recent research has cautioned against use of this assessment tool, opting instead for the “H-measure” [24]. Here we display the analysis using confusion matrices, and provide assessments of model accuracy using the area under the curve and H-measure. Higher H-measure values are associated with more accurate and precise predictive measures.

The risk scoring was first applied to wild poliomyelitis outbreaks in August 2014, and the analyses have been repeated every six months since. Countries with endemic circulation of wild poliovirus were excluded from the risk assessment as SIAs within these countries were determined independently, and details of a risk-based analysis are found elsewhere [25, 26]. For consistency with other assessments within the Global Polio Eradication Initiative (GPEI), countries reporting one or more wild or vaccine-derived poliomyelitis cases were allocated a High classification in the final risk score, and countries identified as “vulnerable” by the Committee of the Public Health Emergency of International Concern [27] were allocated a Medium High risk. The risk scoring method was first applied to cVDPV data in August 2015, and included countries with endemic poliomyelitis. The VDPV risk for July–December 2016 was not considered as the removal of serotype 2 from the OPV formulation in April 2016 will affect the expected risk and accounting for this is beyond the scope of the analysis.

All analyses were carried using R statistical programming (v 3.3.1.).

## Results

### Wild poliomyelitis outbreaks

**Fig. 1:**
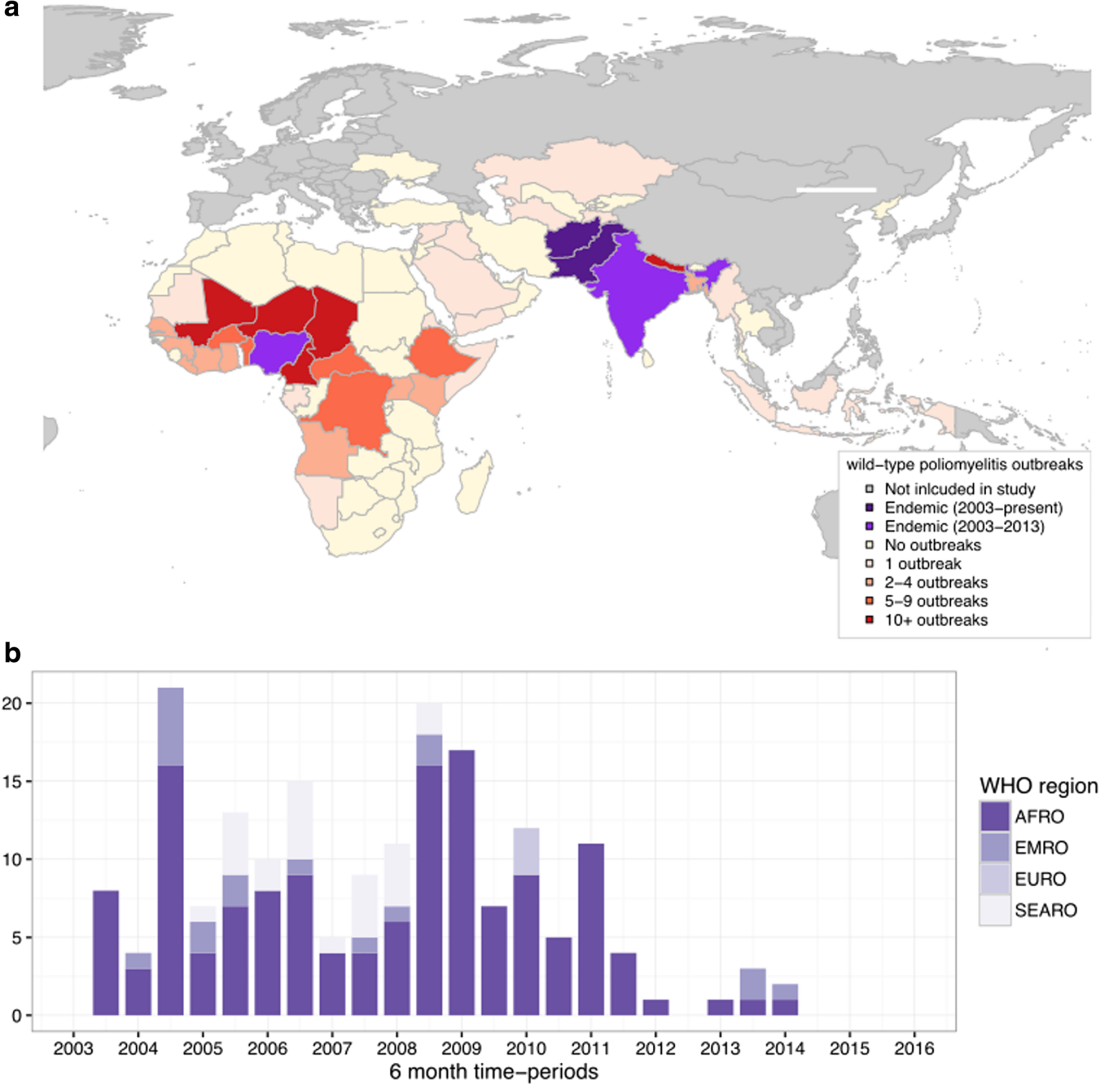
Location (**a**) and timing (**b**) of wild poliomyelitis outbreaks within AFRO, EMRO, SEARO and selected EURO countries, 2003–2015. The publication of this map does not imply the expression of any opinion whatsoever on the part of WHO concerning the legal status of any territory, city, or area or of its authorities, or concerning the delimitation of its frontiers or boundaries

From January 2003 to 30 June 2016, 196 genetically distinct wild outbreaks were reported within the AFRO, EMRO, SEARO regions and selected EURO countries (totalling 92 countries, Fig. 1). There were 150 (76.5% of total) outbreaks of serotype 1, and the remainder were serotype 3; 91 (46.4%) outbreaks consisted of multiple cases and 13 outbreaks continued in excess of 1 year within each country. The number of outbreaks reported per six-month period varied from 0 to 21, with an average of 7.26 outbreaks per six-month period. The timing of outbreaks peaked in the second half of both 2004 and 2008, which at the time corresponded to the peak in incidence of poliomyelitis cases in Nigeria and other countries in Central Africa. No cases were reported in Nigeria since July 2014, until in August 2016 two poliomyelitis cases were reported in Borno State with onset of symptoms in July. These cases were genetically linked to endemic transmission within Nigeria where the last associated case was reported in 2013, meaning that transmission has continued undetected since this time. After 2007 there has been a gradual reduction in outbreaks where the last wild outbreak was reported in Iraq, consisting of two cases where the date of onset of the first case was in February 2014. The last case of wild poliomyelitis outside of the endemic countries was in Somalia in August 2014.

In the multivariate model, factors significantly (*p* < 0.05) associated with an increased probability of a country reporting an outbreak were national DTP3 immunisation estimates below 80%, bordering countries with poliomyelitis cases in the previous 6 months, a high migration rate from countries reporting poliomyelitis cases in the previous 6 months, diarrhoea-associated mortality above 200 cases per 100,000 and experiencing an outbreak in the previous 4 years (Table 2). Additional indicators of population immunity (the percentage of under-immunised and zero-dose non-polio AFP cases above 20%) were also included in the final model even though routine immunisation had the strongest association with wild outbreaks out of the three population immunity measures tested. The national estimate for routine immunisation was updated annually whereas the non-polio AFP measure was estimated every six months and may consequently be more responsive to changes in population immunity. Population displacement was also included in the final risk model although its association with wild poliomyelitis risk was non-significant (*p* = 0.116). The data source for measuring population displacement was updated every six months and may be more responsive to changes in susceptibility, whereas other data sources of migration and susceptibility do not vary in time. Additionally, we identified that the incidence of diarrhoea-associated mortality was confounded with population displacement, and therefore both factors were included in the risk model. Measures of migration that may increase poliovirus exposure and countries with humanitarian emergencies, both which were based on expert opinion, were also included in the final risk model.

**Table 2:** Explanatory variables used in the risk score to assess poliomyelitis outbreak risk, based on a regression analysis of data from 2003 to 2016

Variable	Factor	*p*-value	Risk estimate (95% CI)	Risk score
Population immunity				
Under-immunised	0–20% of non-polio AFP reporting 0–2 OPV doses		baseline	0
	>20% of non-polio AFP reporting 0–2 OPV doses	0.180	1.48 (0.84, 2.61)	1
Zero dose	0–20% of non-polio AFP reporting 0 OPV doses		baseline	0
	>20% of non-polio AFP reporting 0 OPV doses	0.427	0.4 (0.04, 3.82)	1
Routine immunisation	80–100% of children under 2 with 3 DPT doses		baseline	0
	<80% of children under 2 with 3 DPT doses	0.023	2.64 (1.57, 4.45)	1
Exposure to poliomyelitis				
Bordering countries with wild poliomyelitis in last 6 months	No		baseline	0
	Yes	<0.001	4.77 (2.31, 9.88)	1
Migration and wild exposure	Low		baseline	0
	Medium	0.060	1.77 (0.98, 3.2)	1
	High	<0.001	5.58 (2.93, 10.65)	1
Migration and wild poliomyelitis exposure - expert opinion	Limited evidence of wild poliomyelitis exposure		baseline	0
	Evidence of wild poliomyelitis exposure	not tested	not tested	1
Susceptibility				
Population displacement	0–10% of population displaced		baseline	0
	>10% of population displaced	0.116	1.62 (0.89, 2.94)	1
Diarrhoea-associated mortality	0–199 deaths per 100,000 per year in children <5 years		baseline	0
	>200 deaths per 100,000 per year in children <5 years	0.001	1.96 (1.1, 3.52)	1
Previous importations	No wild poliomyelitis importations in previous 4 years		baseline	0
	Wild poliomyelitis importations in previous 4 years	<0.001	2.75 (1.52, 4.97)	1
Humanitarian emergencies - expert opinion	None reported		baseline	0
	Country of concern	not tested	not tested	1

The coefficients of the variables from the regression model were converted into risk scores for each variable, varying from 0 to 1 (Table 2). The risk score for an individual country varied from 0 to 9, which was then translated to a final classification with associated historic probability of experiencing an outbreak (Table 4): Low; 0.8% (95% CI 0.4–1.3), Medium; 5.6% (95% CI 3.1–8.2), Medium High; 18.5% (95% CI 14.3–23.9) and High; 35.2% (95% CI 24.2–46.4). If a High classification was used as a cut-off to identify at-risk countries the model had a sensitivity of 24.2%, but if Medium High and High classifications were used the sensitivity improved to 69.7%, with a reduction in specificity to 86.5%.

**Fig. 2:**
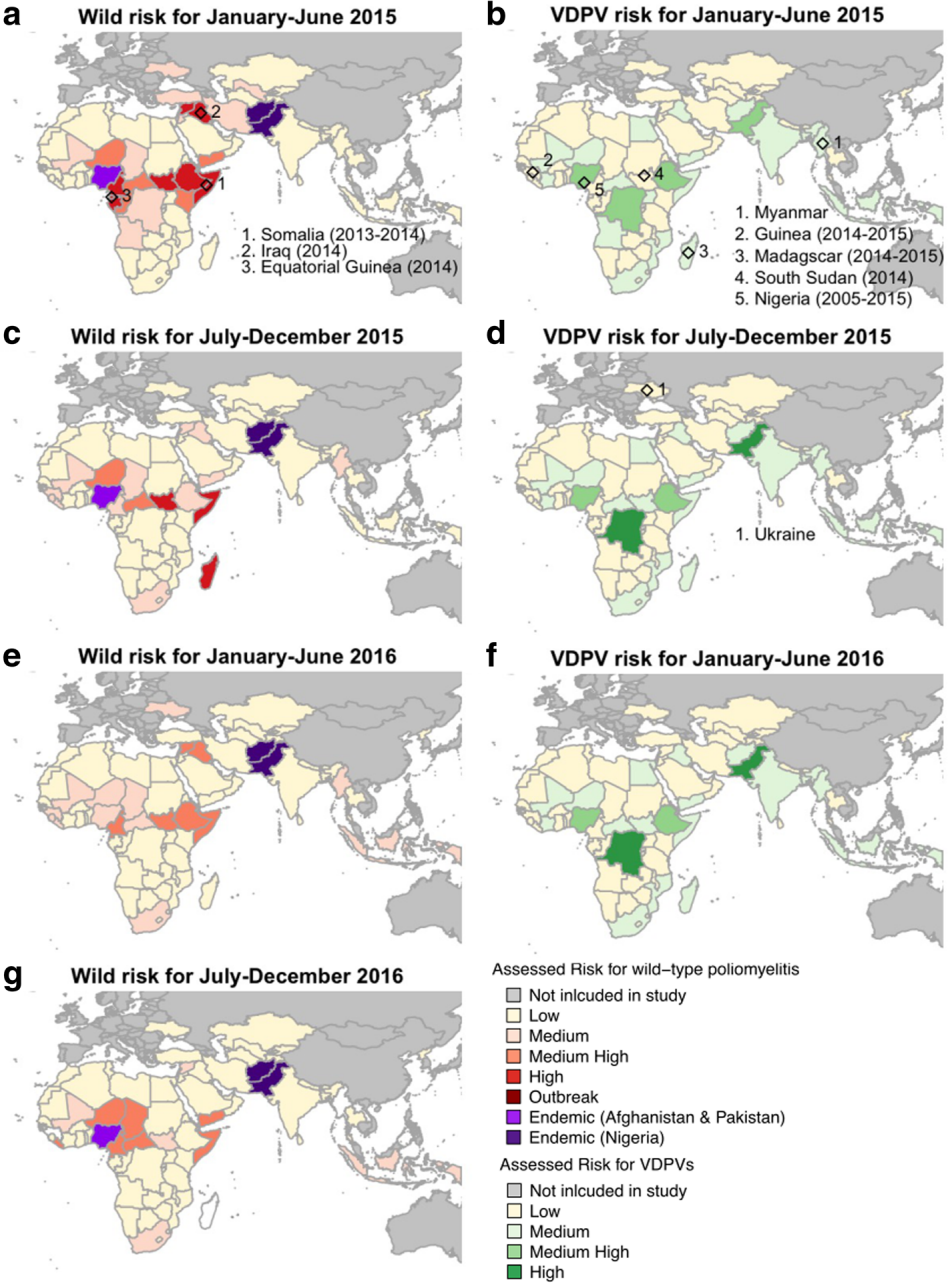
Assessed risk of wild (**a**, **c**, **e**, **g**) and vaccine-derived (**b**, **d**, **f**) outbreaks of poliomyelitis from January–June 2015 to July–December 2016. Confirmed outbreaks of wild and cVDPV within each time-period are also indicated and the parentheses indicate if outbreaks from previous time-periods are on-going. Nigeria was included in the wild risk assessment in January–June 2016 and was re-classified as endemic when the July–December 2016 risk assessment was made. The publication of this map does not imply the expression of any opinion whatsoever on the part of WHO concerning the legal status of any territory, city, or area or of its authorities, or concerning the delimitation of its frontiers or boundaries

The risk score has been used to classify countries in six-month ahead forecasts from July–December 2014 onwards, where the model and variables used to assess the risk have been slightly modified since this time. The predictive ability of the forecasts from the regression model, risk score and classification were assessed using the H-measure; the regression model had a value of 0.453 and the risk score and classification models had values of 0.437 and 0.435 respectively (a ~ 3% reduction). Inclusion of population immunity indicators (under-immunised and zero-dose estimates) was associated with a small (<0.7%) reduction in predictive ability and was not considered to negatively influence the risk score. In the five rounds of risk assessment the number of countries classified as Low has increased from 39.0% to 77.5%, and the number of countries classified as either Medium High or High has decreased from 31.7% to 9.0% (Fig. 2). Within this time-period, two outbreaks were reported in Iraq and Equatorial Guinea, where in the January–June 2014 risk assessment both countries were classified as High. The detection of wild poliomyelitis in August 2016 in Nigeria resulted in increasing the outbreak risk in neighbouring countries. Maps of the risk classification up to July–December 2016 are shown in Fig. 2; Syria, South Sudan, South Africa, Indonesia and Mali were classified as Medium risk and Yemen, Somalia, Niger, Liberia, Chad, Central African Republic, Cameroon and Equatorial Guinea were classified as Medium High.

### Vaccine-derived poliomyelitis outbreaks

**Fig. 3:**
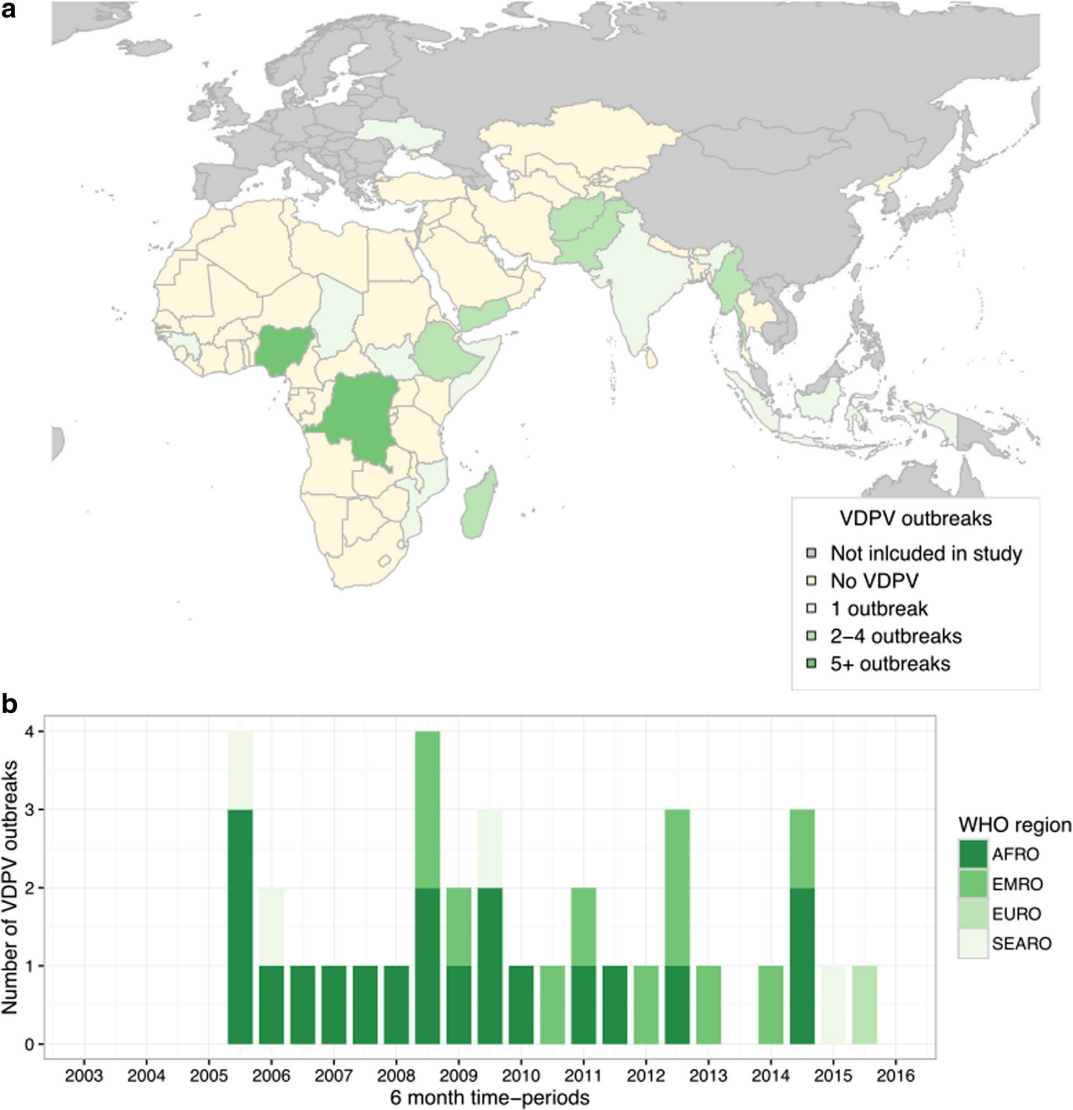
Location (**a**) and timing (**b**) of vaccine-derived poliomyelitis outbreaks within the AFRO, EMRO, SEARO and selected EURO countries, 2003–2016. The publication of this map does not imply the expression of any opinion whatsoever on the part of WHO concerning the legal status of any territory, city, or area or of its authorities, or concerning the delimitation of its frontiers or boundaries

Based on the definition of cVDPVs used in this paper, since 2003 there have been 35 outbreaks of cVDPVs, located in 16 countries (Fig. 3). Nigeria and the Democratic Republic of the Congo (DRC) experienced over ten outbreaks across the time period. A majority (*n* = 28 (80%)) of cVDPV outbreaks were of serotype 2; 5 outbreaks (Indonesia 2005, Myanmar 2006, Mozambique 2011, Madagascar 2014 and Ukraine 2015) were of serotype 1 and 3 outbreaks (Yemen 2011 and 2012, Ethiopia 2009) were of serotype 3. Outbreaks within the Western Pacific Region (reported in China and Lao People’s Democratic Republic) were excluded from this analysis.

In the multivariable model four risk factors were associated with an increased probability of cVDPV outbreaks: the national percentage of children under 2 immunised with DTP3, reporting of cVDPV outbreaks in the previous four years, the percentage of the population displaced and the numbers of children born per year (Table 3). Estimates of the percentage of children under-immunised and reporting zero OPV doses were also included even though their association with cVDPV outbreaks were non-significant (*p* > 0.05). The regression coefficients were used to calculate a risk score, which per variable varied from 0 to 4, resulting in the risk score varying from 0 to 9. The scores were converted into classifications and the probability of experiencing an outbreak, sensitivity and specificity are reported (Table 4). If Medium High and High observations were used to identify countries at-risk of a cVDPV outbreak, this was associated with a sensitivity of 67.6% and specificity of 93.4%, where the sensitivity is slightly lower in comparison to the wild risk assessment. The predictive ability of the regression model, risk score and classification were assessed using the H-measure, the value for the regression model was 0.50 and the risk classification was 0.46 (an 8% reduction).

**Table 3:** Explanatory variables used in the risk score to assess vaccine-derived poliomyelitis outbreak risk, based on a regression analysis of data from 2003 to 2016

Variable	Factor	*p*-value	Risk estimate	Risk score
Population Immunity				
Routine	95+		baseline	0
immunisation	85–95	<0.001	1.51 (1.23, 1.85)	1
	75–85		2.27 (1.85, 2.78)	1
	65–75		3.42 (2.79, 4.19)	2
	<65		5.15 (4.2, 6.31)	2
Under-immunised	0–20% of non-polio AFP reporting 0–2 OPV doses		baseline	0
	>20% of non-polio AFP reporting 0–2 OPV doses	0.18	1.48 (0.84, 2.61)	1
Zero dose	0–20% of non-polio AFP reporting 0 OPV doses		baseline	0
	>20% of non-polio AFP reporting 0 OPV doses	0.427	0.40 (0.04, 3.82)	1
Susceptibility				
cVDPV outbreaks in last 0–4 years	No		baseline	0
	Yes	0.005	3.41 (1.44, 8.07)	1
Population displacement	0–10% of population displaced		baseline	0
	>10% of population displaced	0.005	3.25 (1.43, 7.38)	1
Livebirths per year	<500,000		baseline	0
	<1,000,000	0.004	6.94 (1.86, 25.93)	2
	+1 mill	<0.001	29.32 (8.3, 103.48)	4

**Table 4:** Risk scores used to determine classification of countries according to wild and cVDPV risk and associated sensitivity, specificity and historical probability of an outbreak

Classification	Low	Medium	Medium high	High
Wild-type assessment				
Score from risk factors	0–2	3	4–5	6–8
% of observations associated with an outbreak (95% CI)	0.8 (0.4, 1.3)	5.6 (3.1, 8.2)	18.5 (14.3, 23.9)	35.2 (24.2, 46.4)
Sensitivity	100.0%	89.9%	69.7%	24.2%
Specificity	0.0%	67.9%	86.5%	97.5%
cVDPV-type assessment				
Score from risk factors	0–3	4–5	6–7	8–9
% of observations associated with an outbreak (95% CI)	0.3 (0.1, 0.6)	2.5 (1.2, 4.1)	12.5 (4.1, 22.2)	34.3 (18.2, 51.6)
Sensitivity	100.0%	88.2%	67.6%	38.2%
Specificity	0.0%	73.1%	93.4%	99.0%

Between January–June 2015 to January–June 2016 the Medium High and High VDPV risk were focussed within populous countries and those with low routine immunisation, such as DRC, Ethiopia, Nigeria and Pakistan (Fig. 2), and smaller countries such as Angola, Iraq, Uganda, Yemen and Afghanistan. For the 2016 risk assessment Pakistan, DRC, Ethiopia and Nigeria were allocated a High or Medium High score. Outbreaks have occurred in Guinea, Madagascar, South Sudan and Ukraine when the country was classified as Low in the risk assessment covering these time periods. Within these countries (except Madagascar), DTP3 immunisation was less than 80%, and in Guinea and South Sudan DPT3 was less than 60% during this time but the number of live-births were typically less than 500,000 per year. From one time-period to the next the estimated risk for each country was similar owing to the strong influence of birth numbers on cVDPV risk, and other factors that only change slightly in value between time periods.

## Discussion

We have provided a comprehensive country-level analysis of wild and cVDPV outbreak risk for 92 countries, based on factors that describe population immunity, historical propensity, exposure and susceptibility to poliomyelitis within a country. The modelling provides a framework for assessing wild and cVDPV risk, which allows a robust comparison between countries and time-periods. The risk for wild poliomyelitis has reduced since 2014, particularly in the African continent, where countries classified as High and Medium High have reduced from 31.7% in July–December 2014 to 9.0% in July–December 2016. The risk of VDPVs has remained fairly constant, as shown by the low but consistent number of cVDPV emergent events in time and consistent geographical pattern of at-risk countries.

Wild poliomyelitis risk is associated with low population immunity, increased population movements from countries with wild poliomyelitis and factors that relate to susceptibility. Country-level estimates of routine immunisation had the strongest association with wild type outbreaks when compared to other approximate measures of population immunity, where the variance in routine immunisation was approximately double the variance of the percentage under-immunised via AFP data. Mass immunisation campaigns are designed to raise the percentage of children who have received three or more OPV doses but their impact on preventing outbreaks may be limited by other factors, such as suboptimal SIA coverage, and access to healthcare [28]. The association of diarrhoea-associated mortality with an increased risk of wild poliomyelitis outbreaks is likely to indicate reduced efficacy of the OPV that may be attributed to wider issues associated with interference from other enteroviruses (which may result in diarrhoea) and a high incidence of intestinal pathogens and associated environmental enteropathy [29, 31]. Additionally, the association with diarrhoeal disease may indicate an increased transmission potential owing to climatic factors, poor hygiene and lack of healthcare access [30–32]. Population displacement may result in increased exposure to poliovirus but also provide an indication of a dysfunctional health system. For example estimates of displaced persons of Syrian origin rose from <1% to >13% in 2012 [21] alongside a reduction of DPT3 estimates to <50%, and a wild outbreak was reported in July 2013 [14].

Migration from countries with poliomyelitis cases is a clear driver for wild poliomyelitis outbreaks and can be regarded as a good predictor of risk. Data for international migration was available for only 2001 and reliable time-varying estimates are required [20]. Migration routes are likely to change annually and there are no consistent data sources that capture these changes, consequently we opted to use expert opinion and time-varying data on population displacement within the risk assessment. Since 2013 a majority of poliomyelitis cases on the African continent were part of the West Africa-B1 lineage [33], which suggests that migration from Nigeria had a large impact on poliomyelitis cases in the African continent. Since this time Nigeria has substantially limited transmission of wild poliovirus through heightened surveillance, improvements in population immunity, and a surge in technical capacity within the country [34]. The recent cases in Borno State suggest gaps in surveillance for acute flaccid paralysis and immunisation against poliomyelitis within a region heavily affected by the Boko Haram insurgency [35]. Outbreaks in the African continent within the last 2 years are likely to have been contained and the last reported case of poliomyelitis outside of Nigeria was in Somalia in August 2014. Consequently, exposure to wild poliomyelitis has substantially reduced across the African continent, but as indicated via the risk assessment many countries within Africa remain susceptible to outbreaks owing to gaps in population immunity and susceptibility indicators. While transmission of poliovirus continues, improvements in population immunity and surveillance for cases remain vital. The South Asian (SOAS) lineage of poliovirus, primarily found in Pakistan and Afghanistan, was associated with outbreaks of poliomyelitis in Syria, Iraq [36] and China (not analysed here). There are fewer examples of cross-border transmission of the SOAS lineage, despite there being more recorded migration from Pakistan and Afghanistan to other countries when compared to Nigeria [20]. In countries directly bordering Pakistan and Afghanistan (Iran, China, Turkmenistan, Tajikistan, India) risk mitigation consists of routine immunisation schedules including up to five OPV doses and SIAs to prevent local transmission, resulting in at least 90% of children under 2 reporting >3 OPV doses [37]. It is therefore likely that these mitigation steps have prevented outbreaks of poliomyelitis within these countries.

The risk score has been used to inform the planning of SIAs within the AFRO, SEARO, EMRO and EURO regions. The multi-agency risk classifications are submitted to the Eradication and Outbreak Management Group of the GPEI, whose remit is to direct activities that will support poliovirus detection and interruption [38]. The allocation of SIAs is made according to the perceived risk of wild and cVDPV outbreaks, available vaccines, resources and finances [3]. Other steps to mitigate outbreaks include improving routine immunisation coverage, adapting the routine immunisation schedule to include more than three doses of OPV, inclusion of immunisation teams at transit points in key locations [19], and increased surveillance for cases of AFP [3]. All of these activities require considerable resources and it has been increasingly important to ensure that the limited resources available for polio eradication are placed where they can be most effective. The risk assessment will continue to be carried out until wild poliomyelitis has been eliminated, but the methods may require some adaptation, for example by including environmental wastewater data, serological surveys and surveillance indicators as they become available. The risk assessment has been used by the GPEI to guide prioritisation of countries in the African region for support to improve case detection, SIA quality and outbreak preparedness in 2015 and again in 2016.

cVDPV risk has factors in common with wild outbreaks such as population immunity, population displacement and a previous history of reporting cVDPV outbreaks. The origins of cVDPV outbreaks differ and this changes the associated risk factors and geographical distribution of risk. Countries where over 1 million children are born each year require suitable health systems to immunise these children against poliomyelitis and other diseases, and in these settings it is especially important to ensure at least 80% of children are immunised. In 2010, DRC, Ethiopia, Nigeria, Pakistan, India and Indonesia were reported to have more than a million births and routine immunisation below 80%, indicating large numbers of children who may be at-risk of cVDPVs. It is likely that the risk of cVDPV emergence can be reduced with improved routine immunisation and SIAs [9], but campaigns with good coverage will be critical to halt transmission. As of April 2016, the inactivated polio vaccine and/or monovalent serotype 2 OPV will only be used in response to circulating virus of that serotype. The model could predict risk in populous countries with low routine immunisation, such as DRC and Pakistan, but had a poor ability to identify ahead of time the risk associated with outbreaks in smaller countries such as Guinea, Madagascar, South Sudan and Ukraine. Low sensitivity of the risk score may be improved by including additional information that is not currently routinely available. Expert opinion of the resilience of specific countries to cVDPVs (for example by quickly initiating an outbreak response), information from surveillance reviews, reports on healthcare access within the country, and sub-national estimates of routine immunisation to account for heterogeneity in coverage [9], may provide additional indicators of cVDPV risk. For example, concerns about a reduction in routine immunisation services during the Ebola outbreak in West Africa were reported in early 2015 [39], and DPT3 coverage in Ukraine had been below 80% since 2008 [40, 41]. A systematic way to incorporate these changes in risk is required to improve the accuracy of the risk assessment.

The inherent risk of cVDPVs associated with OPV use means that the gradual withdrawal of the OPV is essential, and this process started in April 2016 by removing the Sabin 2 strain from routine and SIAs and inclusion of the inactivated polio vaccine in routine immunisation [10]. This step will strongly influence the temporal and geographical distribution of VDPV risk as it removes direct exposure to Sabin 2 poliovirus. Long-term, circulation of VDPV2 will reduce as emergence of VDPV progenitors decline. Surveillance for cases of AFP and poliovirus in wastewater samples will continue to be important in countries free from wild poliomyelitis as detection and response of cVDPVs and its progenitors is essential to prevent disease. Further research is required to understand how the distribution and incidence of cVDPVs of each serotype will change in the absence of serotype 2, and the analysis presented here will be updated to inform the surveillance and response activities associated with cVDPVs.

The use of a risk score based on a regression model is advantageous when compared to using the regression model alone. The risks for additional countries and time periods can be rapidly calculated and the reasons for why specific countries have been allocated a score are transparent, which can be advantageous when engaging with stakeholders with limited statistical modelling experience. There was a small reduction in the H-measure from using a risk score instead of the regression model, but we considered this a minimal reduction. Measures of the variation in uncertainty for each assessment are not available when using the risk score, whereas the prediction from the regression analysis includes estimates of uncertainty. For the purposes described here, statistical uncertainty of risk is not formerly considered when used to prioritise SIAs, and so the risk classification approach is the preferred option.

## Conclusions

Use of the scoring system is a transparent and rapid approach to assess country risk of wild and vaccine-derived poliomyelitis outbreaks. Within the time period of the analyses there has been a steep reduction in the number of wild poliomyelitis outbreaks and the reduction in countries classified as High and Medium High risk has reflected this. Vaccine-derived poliomyelitis outbreak risk has remained relatively constant over time but the location of at-risk countries has changed geographically. These findings highlight that many countries remain susceptible to poliomyelitis outbreaks and maintenance or improvement in routine immunisation is vital.

Looking forward, this analysis provides an assessment of risk for guidance of immunisation activities against poliomyelitis based on the available evidence and historical patterns of wild and cVDPV outbreaks. The analysis may also assist with prioritization of poliomyelitis surveillance and social mobilization activities by highlighting countries where further investment is required. As the incidence of poliomyelitis reduces and transmission is stopped within endemic countries, SIAs with the OPV will likely reduce in line with the declining risk [42]. However, it will be important to remain responsive to the risk of outbreaks this framework can be used to inform immunisation activities and ensure that they are proportionate to the perceived risks.

## Additional file

Additional file 1: Table S1. Reported circulating vaccine-derived poliomyelitis outbreaks since 2003, by six-month window of the first reported case, serotype, and source. **Table S2.** Likelihood versus consequence matrix for assessment of population movement reports. **Table S3.** Reported refugees from outbreak and endemic countries (UNHCR mid-2014 data). Countries highlighted in yellow or orange have been given a score of 1 in the risk assessment. (DOCX 53 kb)

## Abbreviations

AFP: Acute flaccid paralysis
cVDPV: circulating vaccine-derived polioviruses
DRC: Democratic Republic of the Congo
DTP3: Diphtheria-tetanus-pertussis
GPEI: Global Polio Eradication Initiative
OPV: Oral polio vaccine
SIAs: Supplementary immunisation activities
SOAS: South Asian
VDPV: vaccine-derived polioviruses
