# Supplementary material for: An assessment of the geographical risks of wild and vaccine-derived poliomyelitis outbreaks in Africa and Asia
Source: BMC Infect Dis. 2017 May 26;17:367. doi: 10.1186/s12879-017-2443-4 (PMC5446690; doi:10.1186/s12879-017-2443-4)
Supplement: Additional file 1: Table S1. — Reported circulating vaccine-derived poliomyelitis outbreaks since 2003, by six-month window of the first reported case, serotype, and source. Table S2. Likelihood versus consequence matrix for assessment of population movement reports. Table S3. Reported refugees from outbreak and endemic countries (UNHCR mid-2014 data). Countries highlighted in yellow or orange have been given a score of 1 in the risk assessment. (DOCX 53 kb) [file 12879_2017_2443_MOESM1_ESM.docx]

# An assessment of the geographical risks of wild and vaccine-derived poliomyelitis outbreaks in Africa and Asia – Additional file 1

Table S1. Reported circulating vaccine-derived poliomyelitis outbreaks since 2003, by six-month window of the first reported case, serotype, and source.

| Country | Six-month time window | Serotype | Cases | WHO region | ISO3 | Source |
| --- | --- | --- | --- | --- | --- | --- |
| CHINA^2^ | 2004.5 | 1 | 2 | - | CHI | [1] |
| CAMBODIA^2^ | 2005.5 | 3 | 2 | WPRO | CAM | [2] |
| DRC^1^ | 2005.5 | 2 | 7 | AFRO | COD | [3] |
| INDONESIA | 2005.5 | 1 | 46 | SEARO | IDN | [4] |
| MADAGASCAR | 2005.5 | 2 | 3 | AFRO | MDG | [2] |
| NIGERIA | 2005.5 | 2 | 3 | AFRO | NGA | [5] |
| MYANMAR | 2006 | 1 | 2 | SEARO | MMR | [6] |
| NIGERIA | 2006 | 2 | 362 | AFRO | NGA | [5] |
| NIGERIA | 2006.5 | 2 | 11 | AFRO | NGA | [5] |
| NIGERIA | 2007 | 2 | 6 | AFRO | NGA | [5] |
| NIGERIA | 2007.5 | 2 | 2 | AFRO | NGA | [5] |
| DRC^1^ | 2008 | 2 | 15 | AFRO | COD | [3] |
| DRC^1^ | 2008.5 | 2 | 5 | AFRO | COD | [3] |
| ETHIOPIA | 2008.5 | 2 | 5 | EMRO | ETH | [7] |
| NIGERIA | 2008.5 | 2 | 9 | AFRO | NGA | [3] |
| SOMALIA | 2008.5 | 2 | 2 | EMRO | SOM | [7] |
| ETHIOPIA | 2009 | 3 | 4 | EMRO | ETH | [7] |
| NIGERIA | 2009 | 2 | 3 | AFRO | NGA | [3] |
| DRC^1^ | 2009.5 | 2 | 5 | AFRO | COD | [3] |
| INDIA | 2009.5 | 2 | 15 | SEARO | IND | [8] |
| NIGERIA | 2009.5 | 2 | 1 | AFRO | NGA | [3] |
| DRC^1^ | 2010 | 2 | 9 | AFRO | COD | [3] |
| AFGHANISTAN | 2010.5 | 2 | 6 | EMRO | AFG | [8] |
| MOZAMBIQUE | 2011 | 1 | 2 | AFRO | MOZ | [9] |
| YEMEN | 2011 | 3 | 12 | EMRO | YEM | [10] |
| CHINA^2^ | 2011.5 | 2 | 3 | - | CHI | [10] |
| DRC^1^ | 2011.5 | 2 | 30 | AFRO | COD | [3] |
| YEMEN | 2012 | 3 | 4 | EMRO | YEM | [10] |
| AFGHANISTAN | 2012.5 | 2 | 15 | EMRO | AFG | [10] |
| CHAD | 2012.5 | 2 | 17 | AFRO | TCD | [10] |
| PAKISTAN | 2012.5 | 2 | 52 | EMRO | PAK | [10] |
| PAKISTAN | 2013 | 2 | 5 | EMRO | PAK | [11] |
| PAKISTAN | 2014 | 2 | 1 | EMRO | PAK | [11] |
| GUINEA | 2014.5 | 2 | 2 | AFRO | GIN | [12] |
| MADAGASCAR | 2014.5 | 2 | 12 | AFRO | MDG | [11] |
| SOUTH SUDAN | 2014.5 | 2 | 3 | EMRO | SSD | [11] |
| MYANMAR | 2015 | 2 | 2 | SEARO | MMR | [12] |
| LAO PEOPLES DEMOCRATIC REPUBLIC^2^ | 2015.5 | 1 | 5 | WPRO | LAO | [12] |
| UKRAINE | 2015.5 | 1 | 2 | EURO | UKR | [12] |

^1^Democratic Republic of the Congo, ^2^ Not analysed here

## Likelihood-versus-consequence matrix for assessment of expert opinion

Expert opinion was used to grade reports of population movements that are unrecorded in the data currently used in the wild risk assessment. The reports typically consist of estimates of population numbers moving from one country (source) to another (sink) with a date and description of the population characteristics. The reports were graded on the likelihood that the reported movements would include individuals infected with wild poliovirus, and the consequence of that movement resulting in transmission within the sink population. The grading resulted in an assessment varying from Low to Very High (Table 2), and if the assessment was High or Very High the sink country was allocated a score of 1.

Table S2. Likelihood versus consequence matrix for assessment of population movement reports.

| Likelihood / Consequence | Negligible | Moderate | Major |
| --- | --- | --- | --- |
| Almost certain | Medium | High | Very High |
| Possible | Low | Medium | High |
| Rare | Low | Low | Medium |

Examples of available data sources include information from the UNHCR, news reports and informal information from field workers within the GPEI. When the refugee data were tested in the regression model there was a non-significant (p>0.05) association with wild poliomyelitis outbreaks and so this data was not routinely included in the risk assessment. A possible explanation for this is that refugee data are known to be reported with error, and the statistics report the total registered refugees and migrants rather than the number of recently registered migrants. Consequently, these and other data were reviewed during each risk assessment round for inclusion as additional sources of migration associated with poliomyelitis risk.

An example of this is given in Table 3 that was part of the January-June 2015 risk assessment. To account for the consequence criteria, only source countries that were endemic for poliomyelitis or had reported wild cases were considered and the sink countries were reviewed considering additional sources of information (see footnotes). Countries that reported more than 500,000 refugees from poliomyelitis-affected countries were allocated a Very High risk. Countries that had more than 100,000 refugees from poliomyelitis-affected countries and for which there were additional sources of recent migration were allocated a High or Very High risk. South Sudan was also allocated a High risk, based upon reports from field workers, and at the time refugee data was not available for use.

Table S3. Reported refugees from outbreak and endemic countries (UNHCR mid-2014 data). Countries highlighted in yellow or orange have been given a score of 1 in the risk assessment.

|  | **Present in** | | | | | | | | | | | | | | | |
| --- | --- | --- | --- | --- | --- | --- | --- | --- | --- | --- | --- | --- | --- | --- | --- | --- |
| **Refugees from** | South Sudan (no data) | Cameroon | Djibouti | Egypt | Ethiopia | India | Iran | Iraq | Jordan | Kenya | Lebanon | Niger | Syria | Turkey | Uganda | Yemen |
| Pakistan |  |  |  |  |  |  |  |  |  |  |  |  |  |  |  |  |
| Afghanistan |  |  |  |  |  | 10,438 | 950,000 |  |  |  |  |  |  |  |  |  |
| Nigeria |  | 17,431^*^^  40,000^¥^ |  |  |  |  |  |  |  |  |  | 8,385^^^  157,000^¥^ |  |  |  |  |
| Somalia | ^ |  | 19,799 | 6,234 | 244,286 |  |  |  |  | 425,741 |  |  |  |  | 15,408 | 234,826 |
| Iraq |  |  |  | 5,458 |  |  | 32,000 |  |  |  |  |  | 146,200^^^ | 15,342 |  |  |
| Syria |  |  |  |  |  |  |  | 220,360 | 645,585 |  | 1,111,076 |  |  | 798,046 |  |  |

^*^ Refugees in Cameroon from Nigeria rose from 7,459 in Jan-2014 to 17,431 mid-2014

^^^ Adjusted up or down based on expert opinion

^¥^ Media information related to Boko Haram (ABC news)

## Measures of predictive ability

Receiver operator characteristic analysis has been previously used to assess the accuracy of model predictions, where the area under the curve (AUC) was estimated using a binormal parametric model. The estimated value of the AUC is equivalent to the probability that a randomly drawn country where an outbreak was detected had a greater predictive value than a randomly drawn country where no outbreak was detected. Whilst used extensively in the medical literature, the AUC suffers from several drawbacks making its use difficult to justify considering the recent availability of other measures. The interpretation of the AUC is not intuitive. Comparisons between different measurements are difficult if the area under the curves for different measures cross. Critically, the AUC is equivalent to measuring performance of classification rules using metrics that depend on the rules being measured (as the value of the AUC depends on the mixture distribution estimated from data). This has been described by Hand [13] as “… if one chose to compare the heights of two people using rulers in which the basic units of measurement themselves depended on the heights.” We consequently explore the H-measure, which was developed by Hand to overcome the disadvantages of the AUC.

The details of calculating the H-measure are provided by Hand [13]. In essence, the severity of misclassification costs (false positive and false negative) is described using a beta distribution instead of the mixture distribution of the data. Typically a beta(2,2) is used meaning that equal weight is given to false positive and false negative results. The interpretation of the estimated values are the same, implying that higher H-measures provide a more accurate prediction of the outcome of interest.

Confusion matrices for assessing predictive ability of the model

| Not bordering Nigeria | | | | | Bordering Nigeria | | | | All countries | | |
| --- | --- | --- | --- | --- | --- | --- | --- | --- | --- | --- | --- |
|  | No outbreak | Outbreak | % outbreaks | No outbreak | | Outbreak | % outbreaks | No outbreak | | Outbreak | % outbreaks |
| Low | 902 | 7 | 1% | 5 | | 0 | 0% | 907 | | 7 | 1% |
| Medium | 204 | 11 | 5% | 14 | | 1 | 7% | 218 | | 12 | 5% |
| Medium High | 92 | 17 | 16% | 24 | | 11 | 31% | 116 | | 28 | 19% |
| High | 22 | 9 | 29% | 5 | | 4 | 44% | 27 | | 13 | 33% |
| Total | 1220 | 44 | 3% | 48 | | 16 | 25% | 1268 | | 60 | 5% |

## References

1. Yan D, Li L, Zhu S, Zhang Y, An J, Wang D, Wen N, Jorba J, Liu W, Zhong G, Huang L, Kew O, Liang X, Xu W: **Emergence and localized circulation of a vaccine-derived poliovirus in an isolated mountain community in Guangxi, China.** *J Clin Microbiol* 2010, **48**:3274–80.

2. Centers for Disease Control and Prevention (CDC): **Update on vaccine-derived polioviruses.** *MMWR Morb Mortal Wkly Rep* 2006, **55**:1093–7.

3. Gumede N, Lentsoane O, Burns CC, Pallansch M, de Gourville E, Yogolelo R, Muyembe-Tamfum JJ, Puren A, Schoub BD, Venter M: **Emergence of vaccine-derived polioviruses, Democratic Republic of Congo, 2004-2011.** *Emerg Infect Dis* 2013, **19**:1583–9.

4. Estívariz CF, Watkins MA, Handoko D, Rusipah R, Deshpande J, Rana BJ, Irawan E, Widhiastuti D, Pallansch MA, Thapa A, Imari S: **A large vaccine-derived poliovirus outbreak on Madura Island--Indonesia, 2005.** *J Infect Dis* 2008, **197**:347–54.

5. Burns CC, Shaw J, Jorba J, Bukbuk D, Adu F, Gumede N, Pate MA, Abanida EA, Gasasira A, Iber J, Chen Q, Vincent A, Chenoweth P, Henderson E, Wannemuehler K, Naeem A, Umami RN, Nishimura Y, Shimizu H, Baba M, Adeniji A, Williams a J, Kilpatrick DR, Oberste MS, Wassilak SG, Tomori O, Pallansch M a, Kew O: **Multiple Independent Emergences of Type 2 Vaccine-Derived Polioviruses during a Large Outbreak in northern Nigeria.** *J Virol* 2013(February).

6. Centers for Disease Control and Prevention (CDC): **Laboratory surveillance for wild and vaccine-derived polioviruses--worldwide, January 2007-June 2008.** *MMWR Morb Mortal Wkly Rep* 2008, **57**:967–70.

7. CDC: **Update on vaccine-derived polioviruses--worldwide, January 2008-June 2009.** *MMWR Morb Mortal Wkly Rep* 2009, **58**:1002–6.

8. CDC: **Update on vaccine-derived polioviruses--worldwide, July 2009-March 2011.** *MMWR Morb Mortal Wkly Rep* 2011, **60**:846–50.

9. CDC: **Update on vaccine-derived polioviruses--worldwide, April 2011-June 2012.** *MMWR Morb Mortal Wkly Rep* 2012, **61**:741–6.

10. Diop OM, Burns CC, Wassilak SG, Kew OM: **Update on vaccine-derived polioviruses - worldwide, July 2012-December 2013.** *MMWR Morb Mortal Wkly Rep* 2014, **63**:242–8.

11. Diop OM, Burns CC, Sutter RW, Wassilak SG, Kew OM: **Update on Vaccine-Derived Polioviruses - Worldwide, January 2014-March 2015.** *MMWR Morb Mortal Wkly Rep* 2015, **64**:640–6.

12. **Polio this week as of 10 August 2016** [http://www.polioeradication.org/Dataandmonitoring/Poliothisweek.aspx]

13. Hand DJ: **Measuring classifier performance: a coherent alternative to the area under the ROC curve**. *Mach Learn* 2009, **77**:103–123.
